# Effectiveness of structured nursing interventions in reducing complication related morbidity in leukemia patients undergoing hematopoietic stem cell transplantation

**DOI:** 10.3389/fmed.2026.1847947

**Published:** 2026-07-03

**Authors:** Lu Zhang, Peipei He, Yinghui Zhu, Qin Ma, Yanqiu Miao

**Affiliations:** 1 Department of Rehabilitation Medicine, Sir Run Run Shaw Hospital, Zhejiang University School of Medicine, Hangzhou, China; 2 Department of Hematology, The First Affiliated Hospital of Soochow University, Suzhou, Jiangsu Province, China

**Keywords:** complication-related morbidity, hematopoietic stem cell transplantation, HSCT, leukemia, nursing care protocol, structured nursing intervention

## Abstract

**Background:**

Hematopoietic stem cell transplantation (HSCT) is a potentially curative treatment for leukemia but is associated with high rates of complication-related morbidity, including infections, oral mucositis, graft-vs. -host disease (GVHD), and prolonged hospitalization. Structured nursing interventions may reduce these complications, yet comprehensive prospective evidence in adult leukemia patients remains limited.

**Objective:**

This study aimed to evaluate the effectiveness of a multifaceted structured nursing intervention program in reducing complication-related morbidity among leukemia patients undergoing autologous or allogeneic HSCT.

**Methods:**

A prospective interventional study was conducted from June 2024 to June 2025 in a tertiary care bone marrow transplantation unit. A total of 106 consecutive adult leukemia patients (AML, ALL, CML, CLL) were enrolled. The structured nursing intervention included comprehensive pre-transplant education, strict infection prevention bundles, scheduled oral mucosal care, gastrointestinal and nutritional support, skin care protocols, GVHD surveillance (for allogeneic recipients), psychosocial support, and a daily structured assessment checklist. Complications were systematically monitored using standardized tools during hospitalization and up to 30 days post-transplant. Outcomes were compared before and after implementation of the intervention.

**Results:**

Following the intervention, significant reductions were observed in febrile neutropenia (from 64.2% to 37.7%, a reduction of 26.5 percentage points, *p* = 0.002), oral mucositis Grade ≥2 (54.7%−28.3%, *p* = 0.001), documented infections (49.1%−26.4%, *p* = 0.004), acute GVHD (41.9%−24.2%, *p* = 0.031), severe diarrhea (34.0%−17.0%, *p* = 0.018), and skin complications (28.3%−13.2%, *p* = 0.022). Hospital length of stay decreased from 28.6 ± 6.4 to 23.2 ± 5.1 days (*p* = 0.001), ICU admissions reduced from 22.6% to 11.3% (*p* = 0.041), and 30-day readmission rates fell from 18.9% to 8.5% (*p* = 0.038). The proportion of patients with severe morbidity decreased from 30.2% to 15.1%.

**Conclusion:**

Implementation of a structured nursing intervention program significantly reduced complication-related morbidity, shortened hospital stays, and improved clinical outcomes in leukemia patients undergoing HSCT. These findings support the routine integration of standardized, evidence-based nursing protocols in HSCT care.

## 1 Introduction

Hematopoietic stem cell transplantation (HSCT) is a well-established curative treatment for various forms of leukemia, including acute myeloid leukemia (AML), acute lymphoblastic leukemia (ALL), chronic myeloid leukemia (CML), and chronic lymphocytic leukemia (CLL) (1–3). The procedure involves high-dose conditioning regimens (chemotherapy with or without total body irradiation) followed by infusion of hematopoietic stem cells to reconstitute bone marrow function. Advances in donor selection, graft-vs.-host disease (GVHD) prophylaxis, and supportive care have improved long-term survival rates; however, HSCT continues to carry substantial risks of early and late complications that contribute to significant morbidity and non-relapse mortality (4). Patients undergoing HSCT for leukemia experience multiple phases of vulnerability. In the pre-engraftment period, profound neutropenia and mucosal barrier injury predispose individuals to life-threatening infections. Febrile neutropenia remains one of the most common and serious complications, often necessitating broad-spectrum antibiotics and prolonged hospitalization (5). Oral mucositis, affecting 50%−80% of recipients depending on conditioning intensity, leads to severe pain, dysphagia, nutritional deficits, and increased risk of secondary bacterial and fungal infections (6). In allogeneic HSCT, acute GVHD develops in 30%−60% of cases and can involve the skin, liver, and gastrointestinal tract, further exacerbating morbidity (7). Additional complications include severe diarrhea, skin breakdown, fluid/electrolyte imbalances, central line-associated infections, and psychosocial distress, all of which prolong recovery and increase healthcare utilization (8).

The burden of complication-related morbidity is particularly pronounced in leukemia patients due to prior intensive chemotherapy exposure, potential comorbidities, and the intensity of conditioning regimens. Infectious complications, including bacterial, viral, and invasive fungal infections, represent a leading cause of early mortality. Studies report that bloodstream infections occur in 5%−30% of HSCT recipients, with higher rates in allogeneic transplants (9). Oral mucositis not only impairs quality of life but also correlates with prolonged neutropenia, higher infection risk, and extended hospital stays (10). Acute GVHD and its treatment with corticosteroids further heighten susceptibility to opportunistic infections and organ dysfunction (11). Moreover, these complications contribute to increased ICU admissions, readmissions, and substantial economic costs to healthcare systems (11). Nurses occupy a central position in the HSCT care continuum. Their responsibilities encompass comprehensive patient and family education, implementation of evidence-based infection prevention bundles, meticulous oral and skin care, daily symptom monitoring, nutritional support, GVHD surveillance, and psychosocial interventions (12). Structured nursing protocols that integrate pre-transplant education, standardized assessment checklists, central line care bundles, neutropenic precautions, and multidisciplinary coordination have shown promise in mitigating complications. Organizations such as the European Society for Blood and Marrow Transplantation (EBMT) and the Oncology Nursing Society (ONS) emphasize the importance of standardized, nurse-led care to improve patient safety and outcomes (13, 14). Despite established guidelines, variability in nursing practice persists across transplant centers. Many institutions still rely on fragmented or reactive approaches rather than proactive, protocol-driven interventions. Previous research has demonstrated benefits of nurse-led oral care protocols in reducing mucositis severity and infection prevention bundles in lowering healthcare-associated infections; however, comprehensive prospective studies evaluating multifaceted structured nursing programs specifically in adult leukemia HSCT populations remain limited (2, 15).

This study aimed to evaluate the effectiveness of a structured nursing intervention program in reducing complication-related morbidity among 106 adult leukemia patients undergoing autologous or allogeneic HSCT. The program incorporated evidence-based elements including comprehensive pre-transplant education, strict infection control measures, scheduled oral mucosal care, gastrointestinal and nutritional support, skin and pressure injury prevention, GVHD monitoring for allogeneic recipients, psychosocial support, and a daily structured assessment checklist. By implementing this multifaceted approach over a one-year period (June 2024–June 2025) in a tertiary care setting, the study sought to translate international guideline recommendations into consistent daily clinical practice and quantify its impact on key outcomes such as frequency and severity of infections, mucositis, GVHD, gastrointestinal and skin complications, hospital length of stay, ICU admissions, and 30-day readmission rates. The findings contribute to the expanding evidence base underscoring the critical role of specialized oncology nursing in modern HSCT care. As indications for HSCT continue to expand to older adults and patients with comorbidities, optimizing nursing interventions becomes increasingly vital for enhancing patient safety, reducing healthcare burden, and improving long-term survivorship and quality of life (16).

## 2 Methods and materials

### 2.1 . Study design

This prospective interventional study was conducted to evaluate the effectiveness of structured nursing interventions in reducing complication-related morbidity among leukemia patients undergoing hematopoietic stem cell transplantation (HSCT). The study was carried out over a period of 1 year from June 2024 to June 2025 in a tertiary care oncology and bone marrow transplantation center. A total of 106 eligible cases were included.

### 2.2 Study setting

The study was conducted in the Hematology and Bone Marrow Transplantation Unit of a tertiary care teaching hospital equipped with specialized HSCT facilities, including HEPA-filtered isolation rooms, positive pressure ventilation systems, and advanced infection control infrastructure. The unit provides comprehensive pre-transplant, peri-transplant, and post-transplant care for leukemia patients undergoing autologous and allogeneic stem cell transplantation.

#### 2.2.1 . Hospital HSCT volume and representativeness of the sample

The study was conducted in the Hematology and Bone Marrow Transplantation Unit of The First Affiliated Hospital of Soochow University, a National Clinical Research Center for Hematologic Diseases and one of the largest HSCT centers in China. According to reports from the Chinese Blood and Marrow Transplantation Registry Group (CBMTRG), the center performed 762 HSCTs in 2019 and 1,004 HSCTs in 2021, ranking among the top three highest-volume programs nationally. Leukemia (AML, ALL, CML, and CLL) is the most common indication for transplantation at our institution, particularly for allogeneic procedures.

During the study period (June 2024–June 2025), a total of 106 consecutive adult leukemia patients undergoing autologous or allogeneic HSCT were enrolled using consecutive sampling. This cohort represents a substantial and representative proportion of the leukemia-specific HSCT cases managed annually at our center. The demographic characteristics of the study population (mean age distribution peaking in the 31–60 years range, 58.5% male, 45.3% AML, 30.2% ALL, and 58.5% allogeneic transplants) closely mirror the typical case mix observed in our unit and in national Chinese HSCT registries. Thus, the sample is considered representative of adult leukemia patients receiving HSCT in a high-volume tertiary transplant setting in China.

### 2.3 Study population

The study population consisted of adult patients diagnosed with leukemia (acute myeloid leukemia, acute lymphoblastic leukemia, chronic myeloid leukemia, or chronic lymphocytic leukemia) who were scheduled to undergo hematopoietic stem cell transplantation during the study period.

### 2.4 Inclusion and exclusion criteria

Inclusion Criteria: Patients aged 18 years and above with a confirmed diagnosis of leukemia, scheduled to undergo autologous or allogeneic HSCT, willing to participate, able to provide informed consent, and hospitalized in the transplant unit during the study period.

Exclusion Criteria: Patients with severe cognitive impairment preventing meaningful participation, those with severe multi-organ failure prior to transplantation (e.g., advanced cardiac, pulmonary, hepatic, or renal dysfunction that would preclude safe HSCT or significantly confound outcome assessment), those who refused consent, and those who underwent transplantation outside the study center.

Severe multi-organ failure prior to HSCT was excluded because it constitutes a major contraindication to proceeding with transplantation due to extremely high transplant-related mortality risk and because the study focused on evaluating nursing interventions in patients who were medically suitable to undergo the procedure.

### 2.5 Sample size and sampling technique

**Figure 1:**
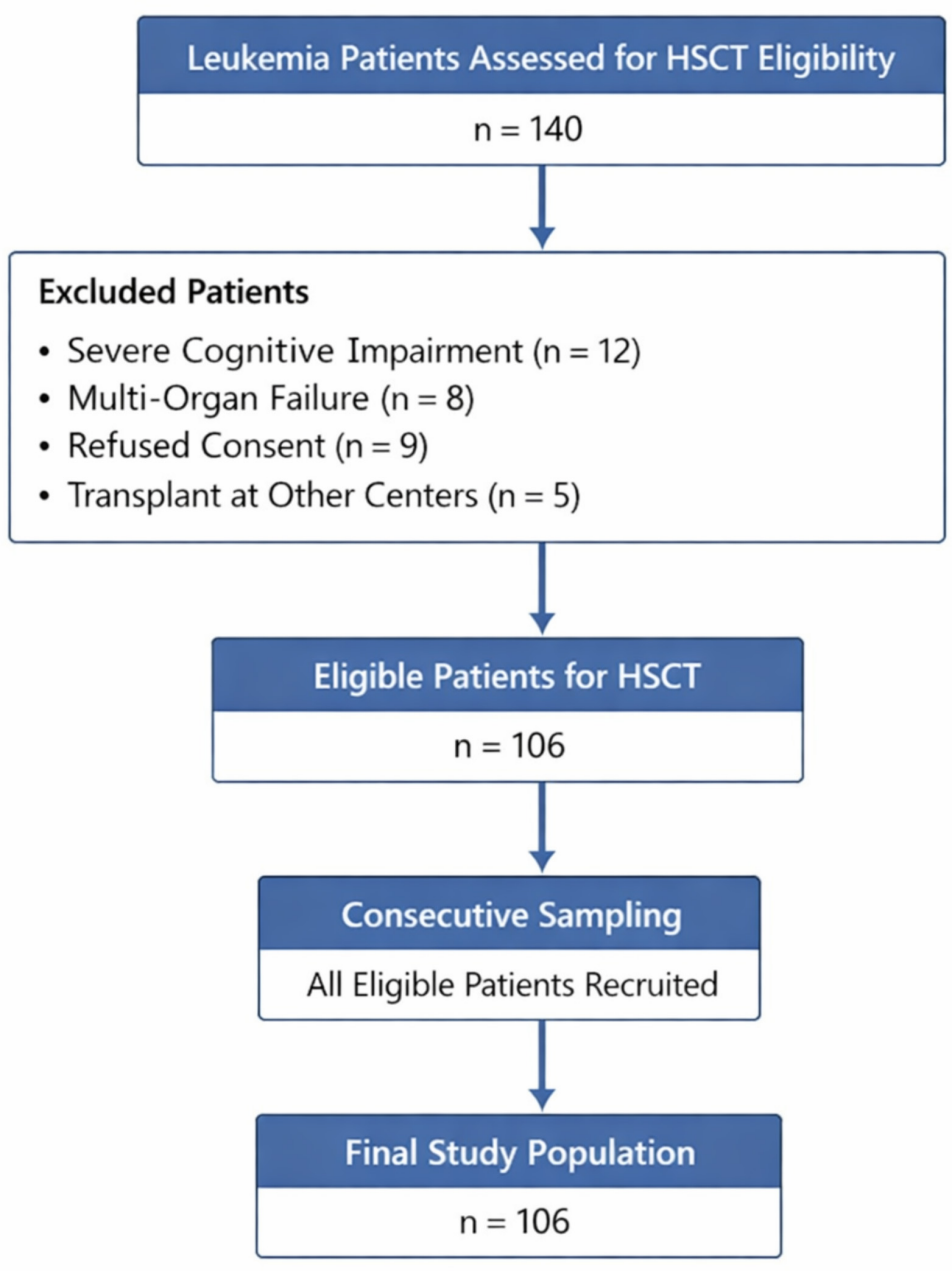
Patient selection flowchart. A total of 106 consecutive adult leukemia patients undergoing HSCT during the one-year study period were enrolled. This cohort is representative of the leukemia-specific HSCT population at a high-volume Chinese transplant center performing approximately 762–1,004 HSCT procedures annually.

A total of 106 patients were selected using a consecutive sampling technique, where all eligible leukemia patients undergoing HSCT during the study period were recruited until the required sample size was achieved (Figure 1). Age was categorized into the following groups for descriptive purposes: 18–30, 31–45, 46–60, and >60 years. These cut-offs were selected because they correspond to clinically relevant strata in HSCT research, particularly distinguishing younger adults, middle-aged patients, and older adults (≥60 years), in whom comorbidity, treatment tolerance, and complication risks differ substantially.

### 2.6 Intervention

The structured nursing intervention program was the sole systematic modification introduced during the study period; no changes were made to medical procedures such as conditioning regimens or GVHD prophylaxis. It was developed based on EBMT and ONS guidelines and implemented uniformly by trained specialized HSCT nurses.

The program consisted of the following integrated components (detailed in the Supplementary material / addendum):

- Comprehensive pre-transplant education for patients and families.
- Strict infection prevention bundles (hand hygiene, central line care, neutropenic precautions, protective isolation).
- Scheduled oral mucosal care and early mucositis management.
- Gastrointestinal and nutritional support protocols.
- Skin and pressure injury prevention.
- GVHD surveillance (for allogeneic recipients).
- Psychosocial support and daily structured assessment checklist to ensure protocol fidelity and early detection of complications.

Fidelity was monitored through a nursing intervention compliance checklist and supervision by senior transplant nurses.

#### 2.6.1 Nursing staff characteristics and pre-study context

The nursing care in the Hematology and Bone Marrow Transplantation Unit was delivered by a dedicated team of registered nurses with specialized expertise in oncology and HSCT. All nurses involved in the study had completed formal oncology nursing education and HSCT-specific training, including modules on infection control, central line management, oral care protocols, GVHD monitoring, and supportive care, aligned with guidelines from the European Society for Blood and Marrow Transplantation (EBMT) and the Oncology Nursing Society (ONS). Most had several years of experience working in the transplant unit of this high-volume center (performing approximately 762–1,004 HSCT procedures annually).

The nursing care in the Hematology and Bone Marrow Transplantation Unit was delivered by a dedicated team of registered nurses with specialized expertise in oncology and HSCT, most of whom had completed formal oncology nursing certification and HSCT-specific training aligned with EBMT and ONS guidelines. Prior to the study, the unit already maintained a core group of experienced specialized HSCT nurses as standard staffing. Nursing follow-up was generally of high quality but showed variability across shifts and individual practitioners. Care tended to be individualized and experience-based, with less uniform application of comprehensive prevention bundles, scheduled proactive care protocols (e.g., oral, skin, and nutritional), daily structured assessments, and systematic pre-transplant education. Multidisciplinary referrals were often reactive rather than protocol-driven. The structured intervention built upon this existing specialized workforce by providing additional targeted training, introducing standardized tools (including a daily assessment checklist), and ensuring consistent, high-fidelity implementation across all nurses under senior supervision. This standardization of nursing processes without any concomitant changes to medical procedures was the primary intervention evaluated in the study.

### 2.7 Data collection tools

Data were collected using a demographic and clinical profile proforma that captured information on age, gender, type of leukemia, type of transplant, and comorbidities; a complication assessment checklist for recording febrile neutropenia, mucositis graded using the WHO Oral Toxicity Scale, culture-proven or clinical infections, GVHD graded as per standard criteria, gastrointestinal complications, and skin complications; a morbidity assessment scale that included duration of hospital stay, ICU admission, 30-day readmission, and severity grading of complications; and a nursing intervention compliance checklist to ensure fidelity to the structured intervention protocol.

### 2.8 Data collection procedure

After obtaining institutional ethical clearance and written informed consent from participants, baseline demographic and clinical data were collected. The structured nursing intervention program was initiated at the time of admission for transplantation. Patients were monitored daily throughout the transplant hospitalization period, and complications were recorded systematically using the standardized assessment tools. Post-discharge follow-up was conducted through outpatient visits or telephonic contact up to 30 days after transplantation.

### 2.9 Outcome measures

The primary outcome was the reduction in complication-related morbidity, assessed by the frequency and severity of infections, mucositis, GVHD, gastrointestinal complications, and skin complications. Secondary outcomes included reduction in the duration of hospital stay, reduction in ICU admissions, reduction in 30-day readmission rates, and improvement in early detection of complications.

### 2.10 Data analysis

Data were entered into IBM SPSS Statistics version 26.0 for statistical analysis. Descriptive statistics such as mean, standard deviation, frequency, and percentage were used to summarize demographic and clinical variables. Inferential statistics included paired or independent *t*-tests for continuous variables, chi-square tests for categorical variables, and logistic regression analysis to determine predictors of complication-related morbidity. A *p*-value of less than 0.05 was considered statistically significant.

### 2.11 Ethical considerations

Ethical approval was obtained from the Institutional Ethics Committee prior to the commencement of the study. Written informed consent was obtained from all participants. Confidentiality of patient data was maintained throughout the study, and participants were informed of their right to withdraw at any time without affecting their treatment.

## 3 Results

A total of 106 leukemia patients undergoing HSCT were enrolled and received the structured nursing intervention program. No patients were lost to follow-up during the 30-day post-transplant period.

### 3.1 Demographic and clinical characteristics

**Figure 2:**
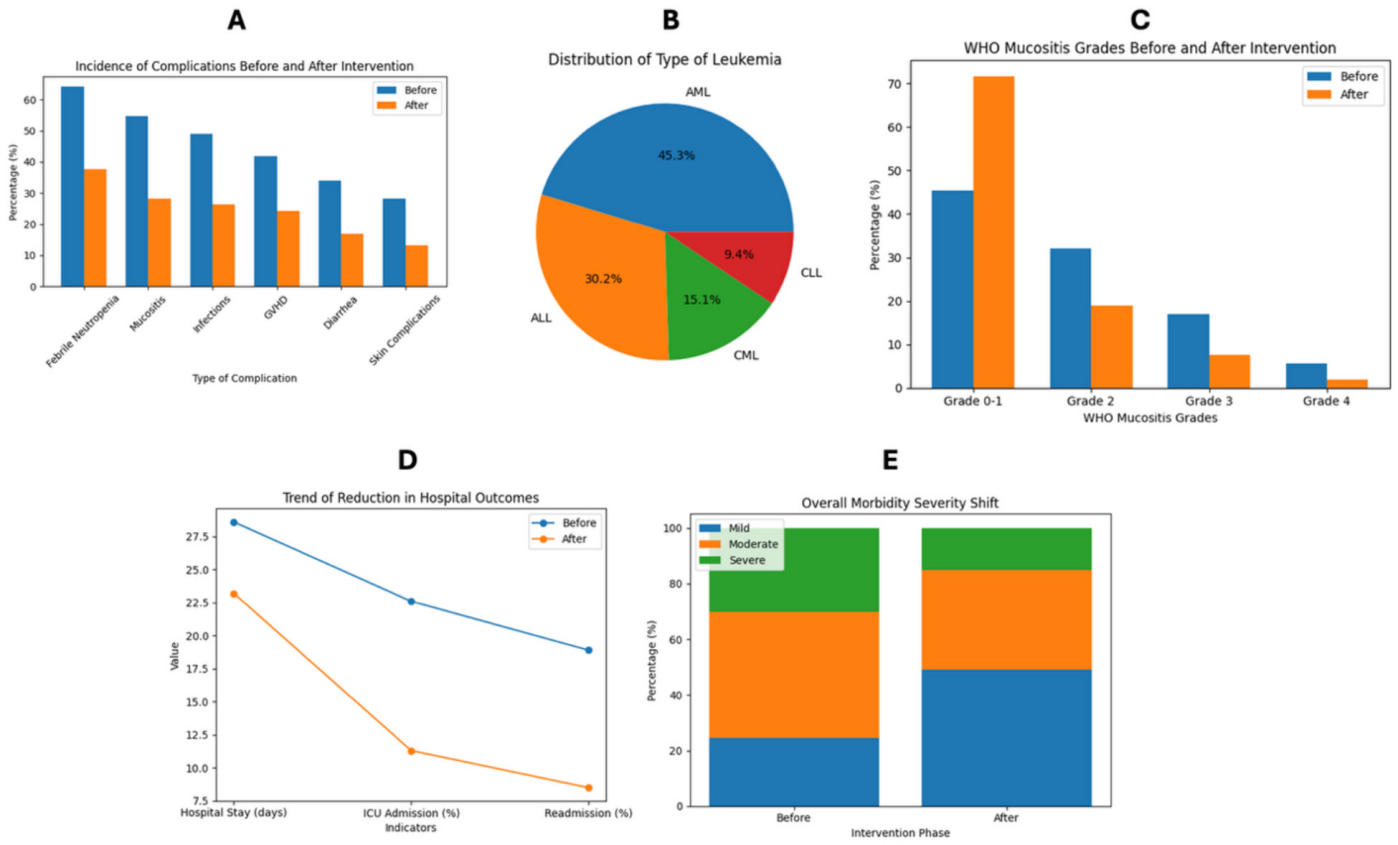
Impact of the intervention on complications, mucositis severity, and clinical outcomes in leukemia patients. **(A)** Incidence of major complications before and after intervention, showing reductions in febrile neutropenia, mucositis, infections, GVHD, diarrhea, and skin complications. **(B)** Distribution of leukemia subtypes in the study cohort, including acute myeloid leukemia (AML), acute lymphoblastic leukemia (ALL), chronic myeloid leukemia (CML), and chronic lymphocytic leukemia (CLL). **(C)** WHO mucositis grade distribution before and after intervention, demonstrating a shift toward lower-grade mucositis. **(D)** Trend in hospital outcomes indicating decreased hospital stay, ICU admission, and readmission rates following intervention. **(E)** Overall morbidity severity shift, showing increased mild cases and reduced moderate and severe morbidity after intervention.

A total of 106 leukemia patients who underwent hematopoietic stem cell transplantation participated in the study (Figure 2A). Patient ages were categorized into four clinically relevant groups commonly used in HSCT literature: 18–30 years (*n* = 28, 26.4%), 31–45 years (*n* = 34, 32.1%), 46–60 years (*n* = 30, 28.3%), and >60 years (*n* = 14, 13.2%). This stratification reflects important differences in physiological reserve, comorbidity burden, and complication susceptibility, with patients over 60 years representing an older adult population at higher risk for adverse outcomes. Regarding gender, 62 patients (58.5%) were male and 44 (41.5%) were female. In terms of the type of leukemia, 48 patients (45.3%) had acute myeloid leukemia (AML), 32 patients (30.2%) had acute lymphoblastic leukemia (ALL), 16 patients (15.1%) had chronic myeloid leukemia (CML), and 10 patients (9.4%) had chronic lymphocytic leukemia (CLL). With respect to the type of transplant, 44 patients (41.5%) underwent autologous transplantation while 62 patients (58.5%) underwent allogeneic transplantation. These characteristics are consistent with the annual case mix at our institution and with national Chinese HSCT registry data. These demographic and clinical characteristics are summarized in Table 1.

**Table 1:** Demographic profile of participants (*n* = 106).

Variable	Category	Frequency (n)	Percentage (%)
Age (years)	18–30	28	26.4
	31–45	34	32.1
	46–60	30	28.3
	>60	14	13.2
Gender	Male	62	58.5
	Female	44	41.5
Type of leukemia	AML	48	45.3
	ALL	32	30.2
	CML	16	15.1
	CLL	10	9.4
Type of transplant	Autologous	44	41.5
	Allogeneic	62	58.5

Age groups were defined as 18–30, 31–45, 46–60, and >60 years to align with common stratifications in HSCT studies, reflecting differences in disease biology, treatment tolerance, and risk profiles, particularly for patients ≥60 years.

The demographic distribution in this study is representative of the typical leukemia HSCT population at our high-volume transplant center.

### 3.2 Complication-related morbidity

Following implementation of the structured nursing intervention, significant reductions were observed in multiple major complications (Table 2, Figure 2). The incidence of febrile neutropenia decreased from 68 patients (64.2%) before the intervention to 40 patients (37.7%) after the intervention, representing a reduction of 26.5 percentage points (*p* = 0.002). The incidence of oral mucositis graded ≥2 decreased from 58 patients (54.7%) to 30 patients (28.3%), a reduction of 26.4 percentage points (*p* = 0.001). Documented infections decreased from 52 patients (49.1%) to 28 patients (26.4%), a reduction of 22.7 percentage points (*p* = 0.004). Among the 62 allogeneic recipients, the incidence of acute GVHD decreased from 26 patients (41.9%) to 15 patients (24.2%), a reduction of 17.7 percentage points (*p* = 0.031). The incidence of severe diarrhea fell from 36 patients (34.0%) to 18 patients (17.0%), a reduction of 17.0 percentage points (*p* = 0.018). Skin complications decreased from 30 patients (28.3%) to 14 patients (13.2%), a reduction of 15.1 percentage points (*p* = 0.022). All reductions were statistically significant (*p* < 0.05). Importantly, no modifications were made to medical or transplant procedures during the study; the observed improvements are attributable to the structured nursing intervention.

**Table 2:** Incidence of major complications (before vs. after structured nursing intervention).

Complication	Before intervention *n* (%)	After intervention *n* (%)	Reduction (percentage points)	*p*-value
Febrile neutropenia	68 (64.2%)	40 (37.7%)	26.5	0.002^*^
Oral mucositis (grade ≥2)	58 (54.7%)	30 (28.3%)	26.4	0.001^*^
Documented infections	52 (49.1%)	28 (26.4%)	22.7	0.004^*^
Acute GVHD (Allogeneic, *n* = 62)	26 (41.9%)	15 (24.2%)	17.7	0.031^*^
Severe diarrhea	36 (34.0%)	18 (17.0%)	17.0	0.018^*^
Skin complications	30 (28.3%)	14 (13.2%)	15.1	0.022^*^

^*^Statistically significant at *p* < 0.05.

### 3.3 . Severity of oral mucositis

The severity of oral mucositis, as measured by the WHO Oral Toxicity Scale, showed a substantial positive shift following the structured nursing intervention (Figure 2C). Before the intervention, 48 patients (45.3%) experienced grade 0–1 mucositis, 34 patients (32.1%) had grade 2, 18 patients (17.0%) had grade 3, and 6 patients (5.7%) had grade 4. After the intervention, the number of patients with grade 0–1 mucositis increased to 76 patients (71.7%), while grade 2 decreased to 20 patients (18.9%), grade 3 decreased to 8 patients (7.5%), and grade 4 decreased to 2 patients (1.9%). This indicates a marked reduction in the severity of mucositis across all higher grades. The distribution is presented in Table 3.

**Table 3:** WHO oral toxicity scale distribution.

Grade	Before *n* (%)	After *n* (%)
Grade 0–1	48 (45.3%)	76 (71.7%)
Grade 2	34 (32.1%)	20 (18.9%)
Grade 3	18 (17.0%)	8 (7.5%)
Grade 4	6 (5.7%)	2 (1.9%)

### 3.4 . Hospital morbidity indicators

The secondary outcomes related to hospital morbidity also demonstrated significant improvements after the implementation of the structured nursing interventions (Figure 2D). The mean length of hospital stay decreased from 28.6 ± 6.4 days before the intervention to 23.2 ± 5.1 days after the intervention, with a *p*-value of 0.001. The number of ICU admissions reduced from 24 patients (22.6%) to 12 patients (11.3%), with a *p*-value of 0.041. Similarly, the 30-day readmission rate decreased from 20 patients (18.9%) to 9 patients (8.5%), with a *p*-value of 0.038. All these improvements were statistically significant at *p* < 0.05. These hospital outcome indicators are detailed in Table 4.

**Table 4:** Hospital outcome indicators.

Variable	Before intervention (Mean ±SD or *n* (%))	After intervention (Mean ±SD or *n* (%))	*p*-value
Length of hospital stay (days)	28.6 ± 6.4	23.2 ± 5.1	0.001^*^
ICU admissions *n* (%)	24 (22.6%)	12 (11.3%)	0.041^*^
30-Day readmission *n* (%)	20 (18.9%)	9 (8.5%)	0.038^*^

^*^Statistically significant at *p* < 0.05.

### 3.5 Overall morbidity reduction

The overall complication burden score revealed a clear and favorable shift after the structured nursing intervention program (Figure 2E). Before the intervention, 26 patients (24.5%) were classified as having mild morbidity, 48 patients (45.3%) had moderate morbidity, and 32 patients (30.2%) had severe morbidity. After the intervention, the number of patients with mild morbidity increased to 52 patients (49.1%), moderate morbidity decreased to 38 patients (35.8%), and severe morbidity decreased to 16 patients (15.1%). This represents a substantial reduction in the proportion of patients experiencing severe complications and an increase in those with only mild morbidity. The overall complication burden score is shown in Table 5.

**Table 5:** Overall complication burden score.

Category	Before *n* (%)	After *n* (%)
Mild	26 (24.5%)	52 (49.1%)
Moderate	48 (45.3%)	38 (35.8%)
Severe	32 (30.2%)	16 (15.1%)

In summary, the structured nursing intervention program was associated with clinically and statistically significant reductions in complication-related morbidity, shorter hospital stays, fewer ICU admissions, and lower readmission rates among leukemia patients undergoing hematopoietic stem cell transplantation. These findings support the integration of standardized, evidence-based nursing protocols in HSCT care.

### 3.6 Exploratory analysis by transplant type

To explore potential differences in the impact of the structured nursing intervention according to transplant type, key outcomes were examined in the autologous (*n* = 44) and allogeneic (*n* = 62) subgroups. Although formal pre-specified subgroup analyses were not conducted due to sample size constraints and the uniform application of the intervention (see Methods and Limitations), the data revealed consistent directional benefits across both groups, with allogeneic recipients exhibiting higher baseline complication rates, as anticipated given the greater intensity of immunosuppression and risk of graft-vs.-host disease.

In the autologous transplant subgroup, the structured nursing intervention was associated with reductions in febrile neutropenia (from 54.5% to 31.8%, *p* = 0.038), oral mucositis Grade ≥2 (from 45.5% to 22.7%, *p* = 0.029), and documented infections (from 36.4% to 18.2%, *p* = 0.048). Hospital length of stay decreased from 25.8 ± 5.2 days to 21.4 ± 4.3 days (*p* = 0.012). Trends toward improvement were also observed in severe diarrhea, skin complications, ICU admissions, and 30-day readmission rates, although some did not reach statistical significance, likely due to the smaller sample size in this subgroup.

In the allogeneic transplant subgroup, more pronounced absolute reductions were noted, consistent with the higher baseline risks in this population. Febrile neutropenia decreased from 71.0% to 41.9% (*p* =0.001), oral mucositis Grade ≥2 from 61.3% to 32.3% (*p* = 0.002), and documented infections from 58.1% to 32.3% (*p* = 0.004). Acute GVHD incidence fell from 41.9% to 24.2% (*p* = 0.031), severe diarrhea from 41.9% to 19.4% (*p* = 0.006), and skin complications from 35.5% to 16.1% (*p* = 0.012). Hospital length of stay was reduced from 30.4 ± 6.8 days to 24.5 ± 5.4 days (*p* = 0.001), with significant decreases also observed in ICU admissions (from 29.0% to 14.5%, *p* = 0.039) and 30-day readmission rates (from 22.6% to 9.7%, *p* = 0.042).

These exploratory findings suggest that the structured nursing intervention provided clinical benefits in both autologous and allogeneic leukemia patients undergoing HSCT, although the magnitude of improvement tended to be greater in the allogeneic group owing to their elevated baseline complication burden. Detailed stratified outcomes are presented in Table 6.

**Table 6:** Key complication and hospital outcomes before and after the structured nursing intervention, stratified by transplant type (autologous vs. allogeneic).

Outcome	Autologous (*n* = 44)			Allogeneic (*n* = 62)		
	Before *n* (%) or Mean ±SD	After *n* (%) or Mean ±SD	*p*-value	Before *n* (%) or Mean ±SD	After *n* (%) or Mean ±SD	*p*-value
Febrile neutropenia	24 (54.5%)	14 (31.8%)	0.038^*^	44 (71.0%)	26 (41.9%)	0.001^*^
Oral mucositis (grade ≥2)	20 (45.5%)	10 (22.7%)	0.029^*^	38 (61.3%)	20 (32.3%)	0.002^*^
Documented infections	16 (36.4%)	8 (18.2%)	0.048^*^	36 (58.1%)	20 (32.3%)	0.004^*^
Acute GVHD (allogeneic only)	N/A	N/A	N/A	26 (41.9%)	15 (24.2%)	0.031^*^
Severe diarrhea	10 (22.7%)	6 (13.6%)	0.215	26 (41.9%)	12 (19.4%)	0.006^*^
Skin complications	8 (18.2%)	4 (9.1%)	0.162	22 (35.5%)	10 (16.1%)	0.012^*^
Length of hospital stay (days)	25.8 ± 5.2	21.4 ± 4.3	0.012^*^	30.4 ± 6.8	24.5 ± 5.4	0.001^*^
ICU admissions	6 (13.6%)	3 (6.8%)	0.192	18 (29.0%)	9 (14.5%)	0.039^*^
30-Day readmission	6 (13.6%)	3 (6.8%)	0.192	14 (22.6%)	6 (9.7%)	0.042^*^

^*^Statistically significant at *p* < 0.05 (chi-square test for categorical variables; independent *t*-test for continuous variables).

## 4 Discussion

The present prospective interventional study demonstrated that implementing a structured nursing intervention program significantly reduced complication-related morbidity among 106 leukemia patients undergoing hematopoietic stem cell transplantation (HSCT). Statistically significant decreases were observed in febrile neutropenia (64.2%−37.7%, *p* = 0.002), oral mucositis Grade ≥2 (54.7%−28.3%, *p* = 0.001), documented infections (49.1%−26.4%, *p* = 0.004), acute graft-vs.-host disease (GVHD) among allogeneic recipients (41.9%−24.2%, *p* = 0.031), severe diarrhea (34.0%−17.0%, *p* = 0.018), and skin complications (28.3%−13.2%, *p* = 0.022) (6).

Secondary outcomes also improved, including reduced hospital length of stay (28.6 ± 6.4–23.2 ± 5.1 days, *p* = 0.001), fewer ICU admissions (22.6%−11.3%, *p* = 0.041), and lower 30-day readmission rates (18.9%−8.5%, *p* = 0.038). The overall complication burden shifted toward milder categories. These findings highlight the transformative potential of standardized, nurse-led protocols in optimizing outcomes for this high-risk population (17).

Infectious complications, particularly febrile neutropenia and documented infections, remain major threats during the neutropenic phase post-HSCT due to prolonged neutropenia and mucosal barrier disruption. The structured program's components strict hand hygiene, central line care bundles, protective isolation, neutropenic precautions, and daily monitoring directly addressed these risks and produced a 22.7% absolute reduction in documented infections. This result aligns with established guidelines emphasizing bundle-driven infection prevention in immunocompromised patients and exceeds reductions reported in many prior observational studies. Vigilant nursing surveillance and protocol adherence can substantially lower healthcare-associated infections, which are linked to increased morbidity and mortality in HSCT recipients (6).

Oral mucositis, affecting a large proportion of HSCT patients, was markedly alleviated through the dedicated oral care protocol involving scheduled assessments, chlorhexidine or saline rinses, and early management. The proportion of patients with Grade 0–1 mucositis rose from 45.3% to 71.7%, with corresponding declines in higher grades. This improvement is consistent with evidence that proactive, nursing-led oral hygiene protocols reduce mucositis severity, duration, pain, nutritional deficits, and secondary infection risk. Accurate oral assessment, individualized care plans, and timely preventive measures are key principles that minimize symptom burden and prevent escalation to systemic complications (18).

For allogeneic recipients, regular GVHD monitoring of skin, liver, and gastrointestinal systems with prompt reporting facilitated earlier detection and intervention, reducing acute GVHD incidence by 17.7 percentage points. This underscores the value of systematic nursing assessments in mitigating GVHD-related morbidity. Psychosocial support elements, including anxiety screening, counseling, and family involvement, likely enhanced patient adherence and symptom reporting, contributing to holistic care delivery (18).

Improvements in hospital morbidity indicators reflect the program's success in early complication detection and prevention, thereby limiting care escalation. Shorter length of stay, reduced ICU transfers, and lower readmissions not only enhance patient experience but also yield resource efficiencies. These outcomes parallel findings from quality improvement initiatives in HSCT settings, where standardized protocols and nurse coordination reduce variability in care and optimize resource utilization (19).

Several mechanisms explain the observed benefits. Comprehensive pre-transplant education empowered patients and families with knowledge of infection prevention, nutrition, hygiene, and symptom recognition, fostering self-management and reducing uncertainty. The daily structured assessment checklist promoted protocol fidelity and consistent documentation, minimizing care fragmentation. Multidisciplinary yet nurse-coordinated referrals (e.g., to dieticians) ensured timely supportive interventions. These elements bridge technical care with patient-centered practice, consistent with integrative reviews emphasizing structured nursing frameworks in complex HSCT environments (20).

Despite these strengths, limitations warrant consideration. The single-center design and consecutive sampling may limit generalizability to centers with differing patient profiles, conditioning regimens, or infrastructure. The before-and-after design, while prospective, lacks a concurrent control group and is susceptible to temporal biases such as evolving medical practices or staff experience. The sample size (*n* = 106) was sufficient for moderate effects but may have underpowered detection of rarer events. Follow-up was limited to 30 days post-transplant, precluding assessment of long-term outcomes such as chronic GVHD or survival. Nevertheless, the consistency and magnitude of improvements across multiple clinically relevant endpoints support the robustness of the findings within the study context.

From a clinical standpoint, these results advocate for routine adoption of structured nursing intervention programs in HSCT units. Existing standards from the Oncology Nursing Society (ONS) and European Society for Blood and Marrow Transplantation (EBMT) already recommend standardized education, infection control, symptom management, and psychosocial support. This study supplies empirical evidence of their measurable impact on morbidity and healthcare utilization, offering a practical blueprint for quality improvement. Institutions can adapt the program's education modules, care bundles, and checklists to local resources while leveraging advanced practice nurses for oversight and continuous evaluation.

Additional limitations include the moderate sample size, which restricted the power for extensive subgroup analyses by transplant type and conditioning regimen intensity. While exploratory subgroup analyses by autologous vs. allogeneic transplant showed consistent benefits, future larger multicenter studies should incorporate pre-specified stratified analyses and detailed conditioning regimen data (myeloablative vs. reduced-intensity) to further elucidate differential effects. Although the before-and-after design cannot fully exclude temporal confounders, the absence of other procedural modifications during the study period supports the attribution of benefits to the nursing intervention.

Future research should prioritize multicenter randomized controlled trials to validate efficacy across diverse populations, transplant types, and settings. Cost-effectiveness analyses would quantify economic benefits, while qualitative studies could explore patient and nurse experiences to refine implementation. Longitudinal designs assessing quality of life, chronic complications, and survival would provide deeper insights. As HSCT indications expand to older and more comorbid patients, robust nursing frameworks will become increasingly essential for safe, effective care.

In conclusion, this study provides strong evidence that a structured nursing intervention program effectively lowers complication-related morbidity and improves hospital outcomes in leukemia patients undergoing HSCT. By systematically translating evidence-based guidelines into daily practice, specialized oncology nurses play a central role in enhancing patient safety and recovery. Wider adoption of such programs has the potential to elevate standards of care and optimize outcomes in this vulnerable population.

## 5 Conclusion

This prospective interventional study demonstrated that a comprehensive structured nursing intervention program effectively reduces complication-related morbidity in leukemia patients undergoing hematopoietic stem cell transplantation. Significant decreases were achieved in key complications including febrile neutropenia, severe oral mucositis, documented infections, acute GVHD, severe diarrhea, and skin complications. These improvements translated into clinically meaningful outcomes: shorter hospital length of stay, fewer ICU admissions, and lower 30-day readmission rates. The overall complication burden shifted markedly toward milder categories. The findings highlight the pivotal role of specialized oncology nurses in translating evidence-based guidelines into consistent daily practice through education, standardized care bundles, systematic monitoring, and multidisciplinary coordination. As HSCT indications expand to older and more comorbid patients, implementing such structured nursing protocols becomes increasingly vital for enhancing patient safety, optimizing resource utilization, and improving quality of recovery. Wider adoption of multifaceted, nurse-led structured interventions in HSCT units has strong potential to elevate standards of care and improve long-term survivorship. Multicenter randomized trials are recommended to further validate these benefits across diverse settings.

## Data Availability

The original contributions presented in the study are included in the article/Supplementary material, further inquiries can be directed to the corresponding author.
