# Supplementary material for: Effectiveness of structured nursing interventions in reducing complication related morbidity in leukemia patients undergoing hematopoietic stem cell transplantation
Source: Front Med (Lausanne). 2026 Jul 3;13:1847947. doi: 10.3389/fmed.2026.1847947 (PMC13377357; doi:10.3389/fmed.2026.1847947)
Supplement: Supplementary file 1 [file Table_1.docx]

**Supplementary Material S1: Structured Nursing Intervention Program Guidelines and Template for Leukemia Patients Undergoing HSCT**

**1. Program Overview** Multifaceted, nurse-led protocol applied from admission through 30 days post-transplant. Goal: Reduce preventable complications via standardization, proactive care, and patient empowerment. Delivered by specialized HSCT nurses with ongoing training and daily checklist supervision.

**2. Key Components and Timeline**

**Pre-Transplant Phase (upon admission / before conditioning)**

- **Education Module**: 1–2 sessions (30–60 min each) covering HSCT process, infection risks, hygiene, nutrition, symptom recognition (fever, mucositis, GVHD signs), and self-management. Use teach-back method; provide written materials and family involvement.
- **Baseline Assessment**: Demographic/clinical profile, oral exam (WHO scale), skin assessment, nutritional status, psychosocial screening (anxiety/depression).
- **Central Line Care Bundle Initiation**: Chlorhexidine dressing, daily inspection, hub disinfection.

**Peri- and Immediate Post-Transplant Phase (Hospitalization)**

- **Infection Prevention Bundle** (daily):
  - Strict hand hygiene (before/after all patient contact).
  - Neutropenic precautions: HEPA-filtered room, limited visitors, no fresh flowers/plants.
  - Central line care per bundle (daily chlorhexidine bath if feasible).
  - Daily vital signs + temperature monitoring q4–6h; prompt blood cultures if febrile.
- **Oral Mucosal Care** (scheduled 4–6 times/day):
  - Gentle brushing with soft toothbrush + fluoride toothpaste (if platelets allow).
  - Rinses: Normal saline or sodium bicarbonate (every 2–4 hours while awake); chlorhexidine if tolerated.
  - Early mucositis management: Pain assessment (WHO Oral Toxicity Scale), topical analgesics, ice chips (cryotherapy for melphalan-containing regimens), nutritional support.
  - Daily oral assessment documented on checklist.
- **Gastrointestinal & Nutritional Support**:
  - Daily monitoring of nausea, vomiting, diarrhea, oral intake, weight, and fluid balance.
  - Dietary modifications (low-microbial diet); early dietitian referral.
  - Antiemetics and antidiarrheals per protocol; parenteral nutrition if needed.
- **Skin & Pressure Injury Prevention**:
  - Daily skin assessment (especially palms, soles, pressure points).
  - Repositioning q2h, moisture management, emollients.
  - Avoidance of trauma; sun protection education.
- **GVHD Surveillance (Allogeneic only)**:
  - Daily assessment of skin (rash), gastrointestinal (diarrhea volume/stool consistency), liver (bilirubin, jaundice).
  - Use standardized grading (e.g., Glucksberg or NIH criteria for acute GVHD).
  - Prompt reporting of changes to medical team.
- **Psychosocial Support**:
  - Daily screening for anxiety, distress, sleep issues.
  - Counseling sessions, family education, referral to psychology/social work as needed.

**Daily Structured Assessment Checklist** (core tool for fidelity): Nurses complete and document once per shift (or more if unstable):

- Vital signs & fever
- Oral cavity (WHO grade + pain)
- Skin integrity & GVHD signs
- GI symptoms & nutrition/fluid balance
- Central line site & infection signs
- Psychosocial status
- Compliance with education bundles
- Any new symptoms or deviations → escalate per protocol

**Post-Discharge (up to 30 days)**:

- Outpatient/telephonic follow-up: Symptom review, adherence check, early readmission prevention.
- Reinforcement of self-care (hygiene, oral care, fever management).

**3. Training and Fidelity**

- All participating nurses received additional protocol-specific training sessions prior to implementation.
- Compliance monitored via checklist audit (target >95% adherence).
- Senior nurse oversight and weekly team reviews.

**4. References for Development**

- EBMT Nurses Group recommendations and EBMT Handbook (nursing chapters).
- ONS HSCT nursing standards.
- CDC/ASCT guidelines for infection prevention in HSCT.
- WHO Oral Toxicity Scale and standard GVHD assessment tools.

This template was applied uniformly to all 106 patients. Centers may adapt timing, specific products (e.g., rinse solutions), or add local resources while preserving core bundle elements.

We believe this addendum fully addresses the reviewer’s request and allows other institutions to implement or further test similar programs.
